# Pre-procedural simulation of the right atrium to left ventricle puncture through the inferoseptal process using cardiac computed tomography

**DOI:** 10.1016/j.hrcr.2025.05.016

**Published:** 2025-05-23

**Authors:** Yuichiro Miyazaki, Shumpei Mori, Stefan Bertog, Kalyanam Shivkumar, Venkatakrishna Tholakanahalli

**Affiliations:** 1 David Geffen School of Medicine at University of California Los Angeles, Los Angeles, California; 2 Division of Cardiology, University of Minnesota, Minneapolis, Minnesota; 3 Division of Cardiology, Minneapolis VA Health Care System, Minneapolis, Minnesota

**Keywords:** Catheter ablation, Computed tomography, Inferior pyramidal space, Inferoseptal process, Right atrium to left ventricle puncture, Ventricular tachycardia

## Introduction

The right atrium to left ventricle (RA-LV) puncture is one of the limited options to access the LV in patients after aortic and mitral valve replacement with mechanical valves.^1^, ^2^, ^3^ For the successful approach and to avoid potential complications, pre-procedural planning based on detailed anatomical knowledge is fundamental, even more so as this approach is yet to be a common procedure in many institutions. Here, we share a case of ventricular tachycardia originating from the LV; the patient had a history of aortic valve replacement with a mechanical valve and transcatheter edge-to-edge mitral valve repair. We selected the RA-LV access, guided by detailed three-dimensional (3D) simulation using pre-procedural cardiac computed tomography (CT). We believe pre-procedural cardiac CT offers a unique and special opportunity for customized pre-procedural planning, and it will eventually help clinicians perform this approach with confidence by reducing safety concerns.

## Case report

**Figure 1:**
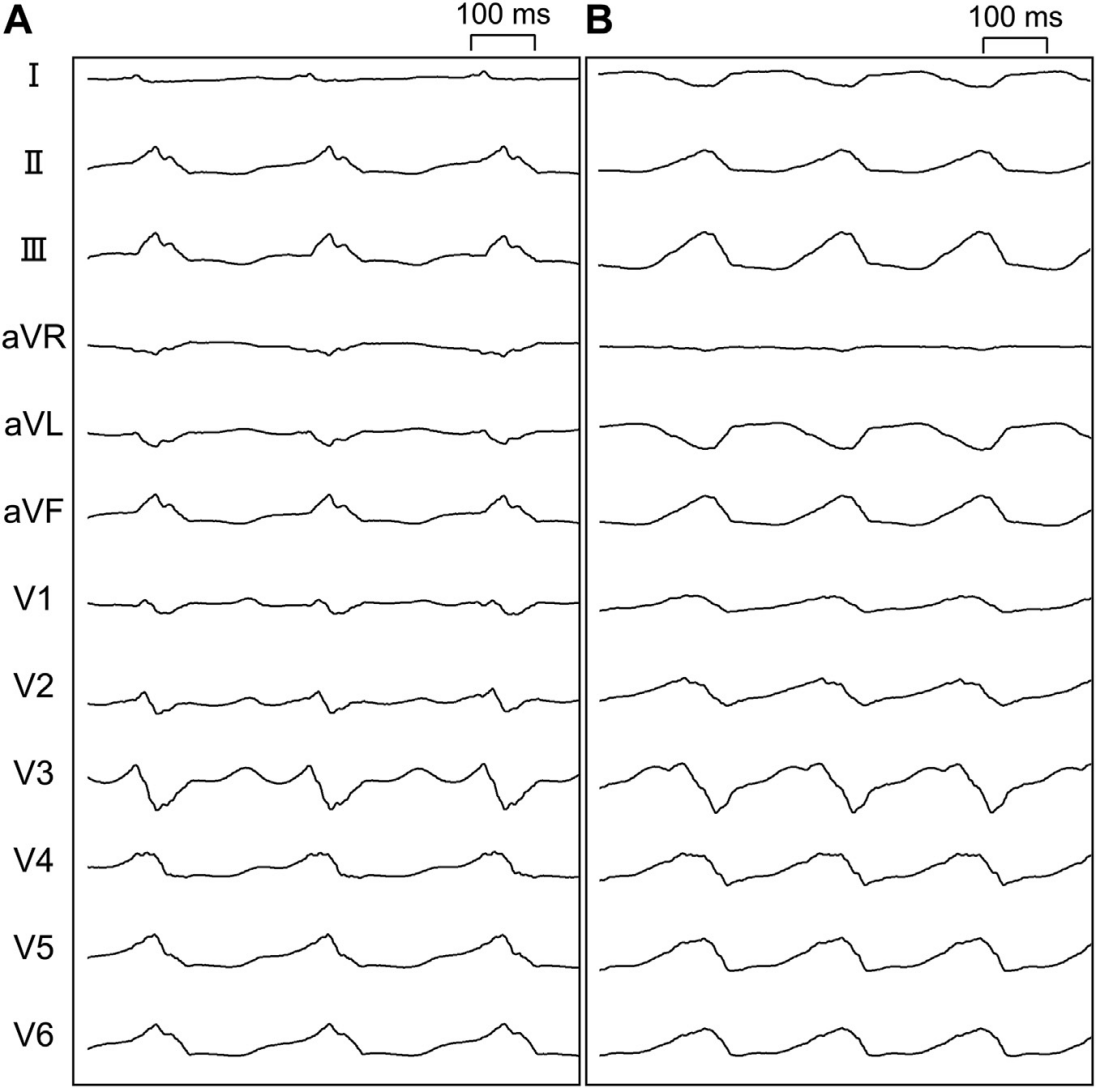
Electrocardiograms during ventricular tachycardia. **A:** The electrocardiogram of ventricular tachycardia 1 shows 536 ms of cycle length with W configuration in V1, left inferior axis, and r wave in lead I, indicating the basal anteroseptal origin. **B:** The electrocardiogram of the ventricular tachycardia 2 shows 425 ms of cycle length with R wave in V1, right inferior axis, and QS wave in lead I, indicating the basal anterior origin.

A 68-year-old male patient with non-ischemic cardiomyopathy presented for catheter ablation for drug-resistant ventricular tachycardia originating from the LV (Figure 1). He had a history of hypertension, dyslipidemia, diabetes mellitus, gout, obstructive sleep apnea syndrome, and chronic obstructive pulmonary disease. He had also undergone catheter ablation for left atrial and left ventricular tachycardias, cardiac resynchronization therapy with defibrillator implantation (left ventricular lead placed at the bundle of His region), aortic valve replacement with a mechanical prosthesis because of aortic root aneurysm or aortic regurgitation, and transcatheter edge-to-edge mitral valve repair for severe mitral regurgitation. His medications included amiodarone, warfarin, acetylsalicylic acid, lisinopril, and allopurinol. Transthoracic echocardiography showed severely reduced left ventricular ejection fraction (27%), increased left ventricular end-systolic (177 mL) and end-diastolic volume (224 mL), and reduced cardiac output (2.8 L/min). To access the LV, the retrograde trans-aortic or antegrade atrial transseptal approaches were deemed unsuitable because of the potential physical interaction between the ablation system and prosthesis in each aortic and mitral valve. The epicardial approach was also withheld because of potential post-surgical adhesions in the pericardial space. Therefore, the RA-LV puncture was selected.

**Figure 2:**
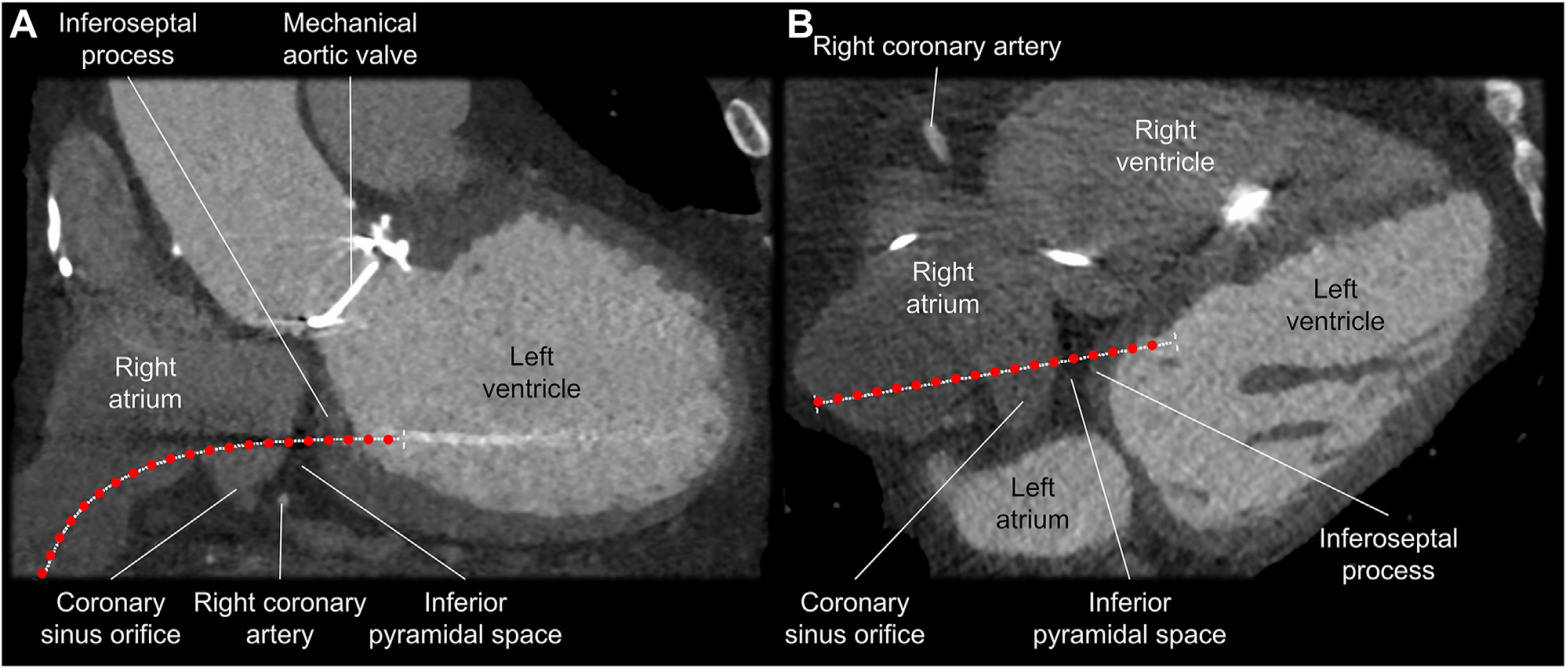
Multiplanar reconstruction images of multidetector-row computed tomography (**A:** coronal, **B:** transverse). The expected three-dimensional route (*red dotted line*) of the right atrium to left ventricle puncture was drawn on the multiplanar reconstruction images of computed tomography.

**Figure 3:**
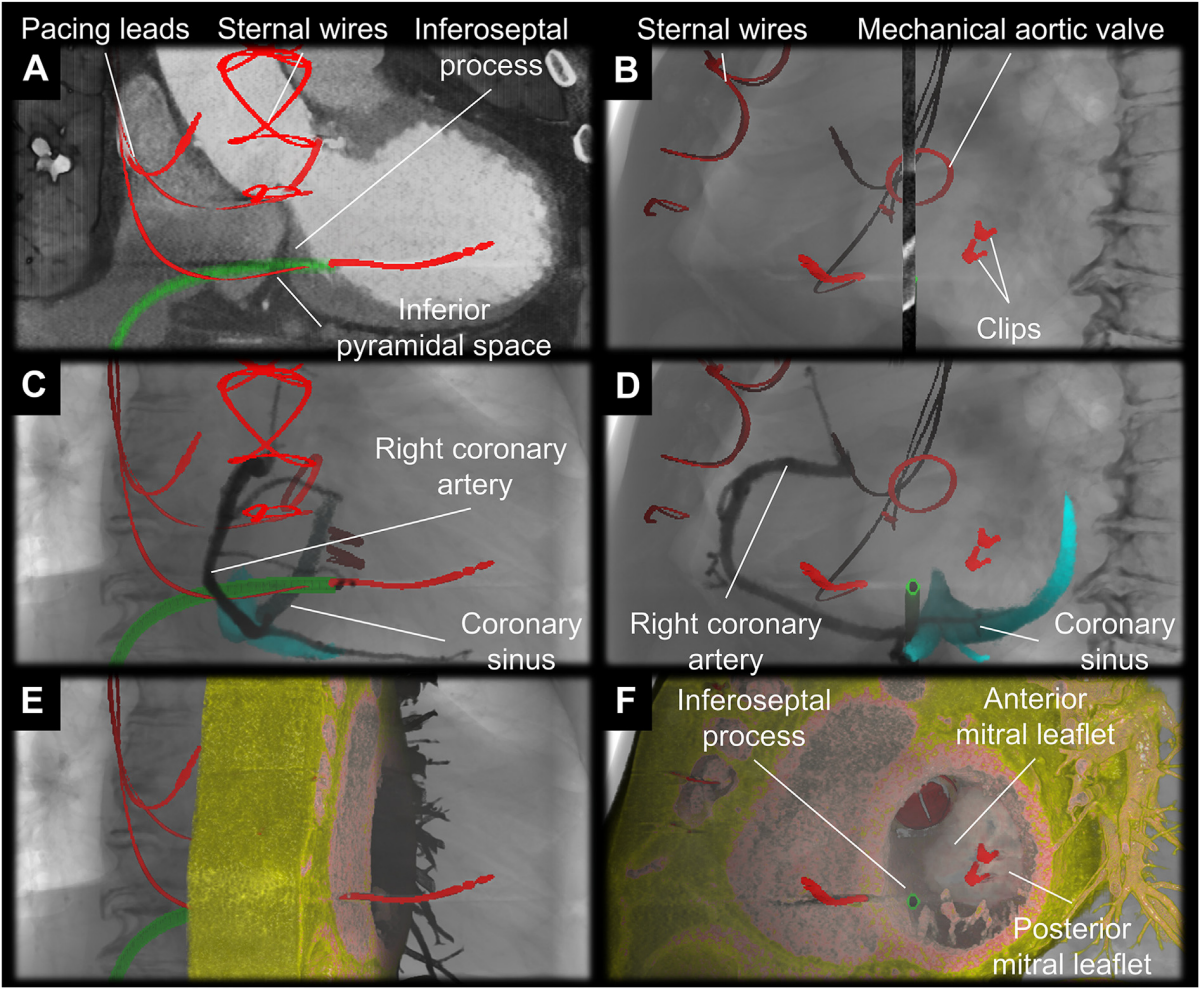
Volume-rendered reconstruction of the virtual sheath viewed from the en-face and tangential directions. **A, C, E:** Course of the virtual sheath (*green*) is viewed from the en-face direction (right anterior oblique 10˚) and **B, D, F:** tangential direction (left anterior oblique 80˚). Within the virtual fluoroscopic image, the sternal wires, pacing leads, mechanical aortic valve and mitral clips (*red*) are reconstructed together with a multiplanar reconstruction plane involving the virtual sheath (**A, B**), with the right coronary artery (*black*) and the coronary sinus (*sky*-*blue*) (**C, D**), and with the short axis cut slab of the heart (**E, F**).

As a part of the preoperative evaluation, electrocardiogram-gated contrast-enhanced CT was performed with a computed tomographic scanner (SOMATOM Force, Siemens Healthineers, Erlangen, Germany). Images reconstructed at mid-diastole (79% of R-R interval) were used for analysis using a commercially available workstation (Ziostation2 version 2.9.8.5; Ziosoft Inc, Tokyo, Japan). First, the optimal path from the RA-LV was estimated by using multiplanar reconstruction images and a 3D path function (Figure 2). As the path must penetrate the epicardial adipose tissue sandwiched between the RA and LV, also referred to as the inferior pyramidal space, it was carefully designed not to interact with coronary arteries observed within the inferior pyramidal space, such as the distal right coronary artery and atrioventricular node artery (Figure 2). Fortunately, in this case, the estimated risk of injury to the right coronary artery was low given its course within the inferior pyramidal space (Figure 2, Supplemental Movie 1). The atrioventricular node artery could not be detected. Then, the path was transformed into a virtual sheath placed in the LV via the RA. Important landmark structures, including the coronary artery and coronary sinus, were additionally reconstructed in a volume-rendered image with extracardiac and intracardiac radiopaque structures, such as sternal wires, clips at the mitral valve, and pacing leads (Figure 3, Supplemental Movie 1). As these artificial structures can be observed under fluoroscopy, they can be used as a marker to guide the procedure. The initial multiplanar reconstruction plane used to draw a path was also visualized on the volume-rendered image to indicate the en-face and tangential view of the plane involving the virtual sheath (Figure 3). By rotating these volume-rendered images (Supplemental Movie 1), optimal biplane angulations of the image intensifier, which was defined as the angulation to observe the virtual sheath in an en-face and tangential fashions, were determined. Because of the significant leftward rotation of the heart of this patient, this pre-procedural simulation recommended a combination of the right anterior oblique 10˚ and left anterior oblique 80˚ as the angulation to guide the procedure (Figure 3).

The procedure was initiated with general anesthesia. Two ventricular tachycardias were induced with programmed stimulation. The first one was 536 ms of cycle length with W configuration in V1, left inferior axis, and r wave in lead I. The other one was 425 ms of cycle length with R wave in V1, right inferior axis, and QS wave in lead I, which was the clinical one (Figure 1). Thus, the substrates and exits of these ventricular tachycardias were estimated around the basal anteroseptum to the anterior wall of the LV, respectively.

**Figure 4:**
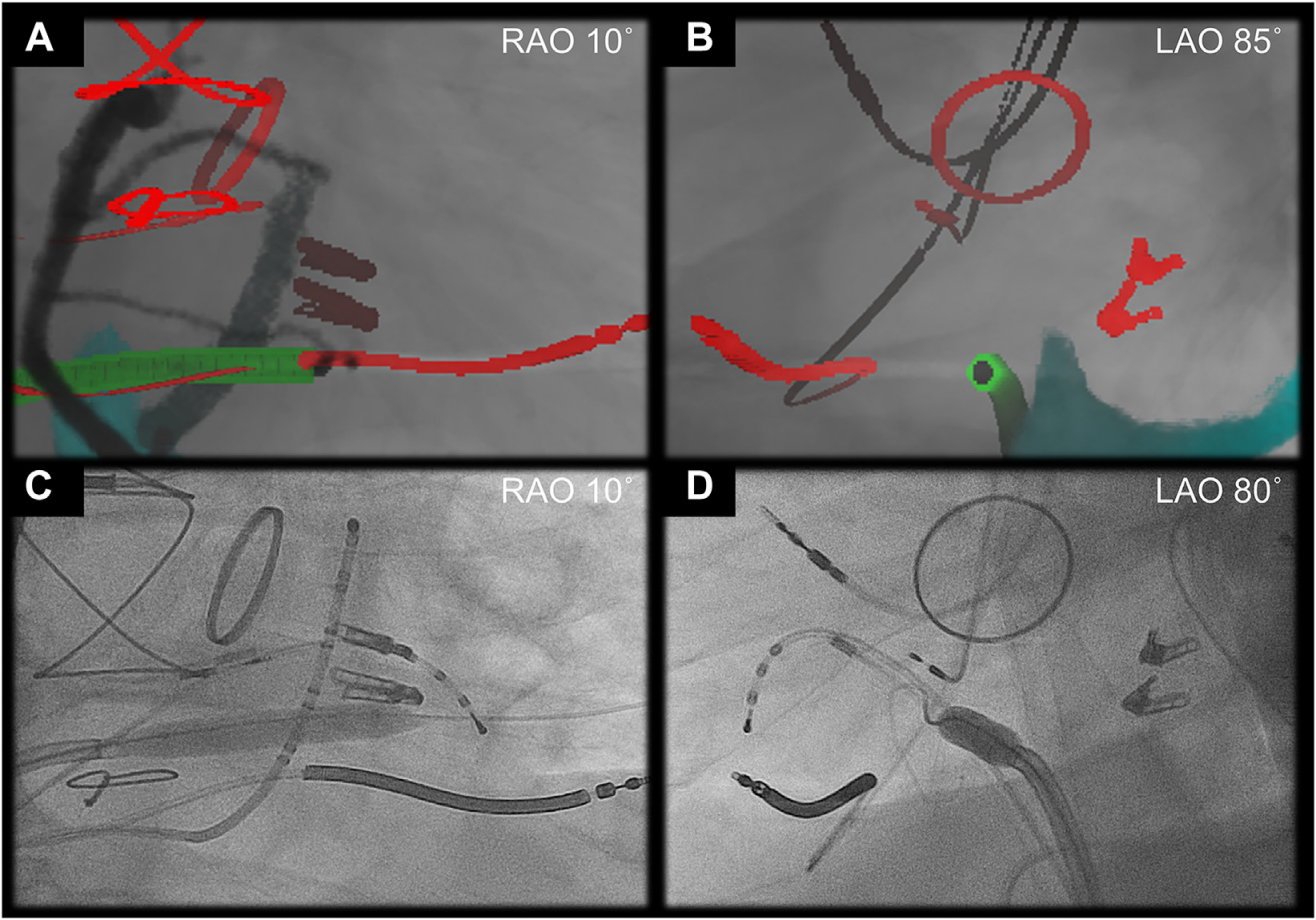
Clinical procedure. Actual procedure was safely performed under the biplane fluoroscopy (**C, D**) using the biplane angulation as simulated by pre-procedural computed tomography (**A, B**). A virtual sheath is reconstructed in green. Approximately 5˚ difference was noted between the computed tomographic simulation and real fluoroscopic image, presumably reflecting the rotational difference of the patient position on the table. In addition, intracardiac markers are more reliable than extracardiac markers as they are not affected by respiration. LAO = left anterior oblique; RAO = right anterior oblique.

**Figure 5:**
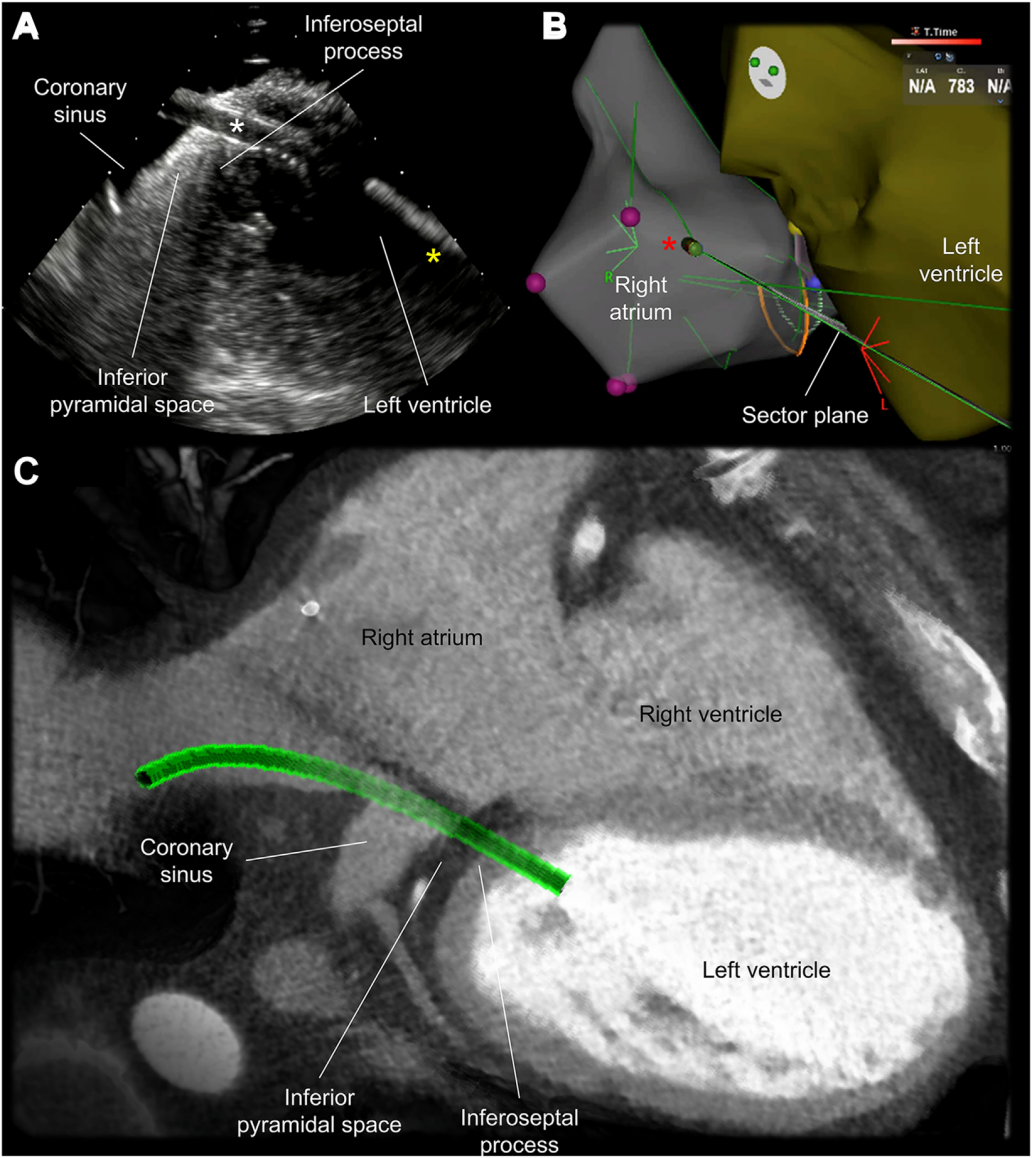
Intracardiac echocardiography. **A, B:** The procedure was performed with real-time assistance of an intracardiac echocardiography. The *white and yellow asterisks* indicate a sheath passing from the right atrium to left ventricle and a catheter placed in the left ventricle, respectively. The *red asterisk* denotes the intracardiac echocardiography probe. **C**: replicates the sectional plane of the intracardiac echocardiography (**A**) with a virtual sheath (*green*).

**Figure 6:**
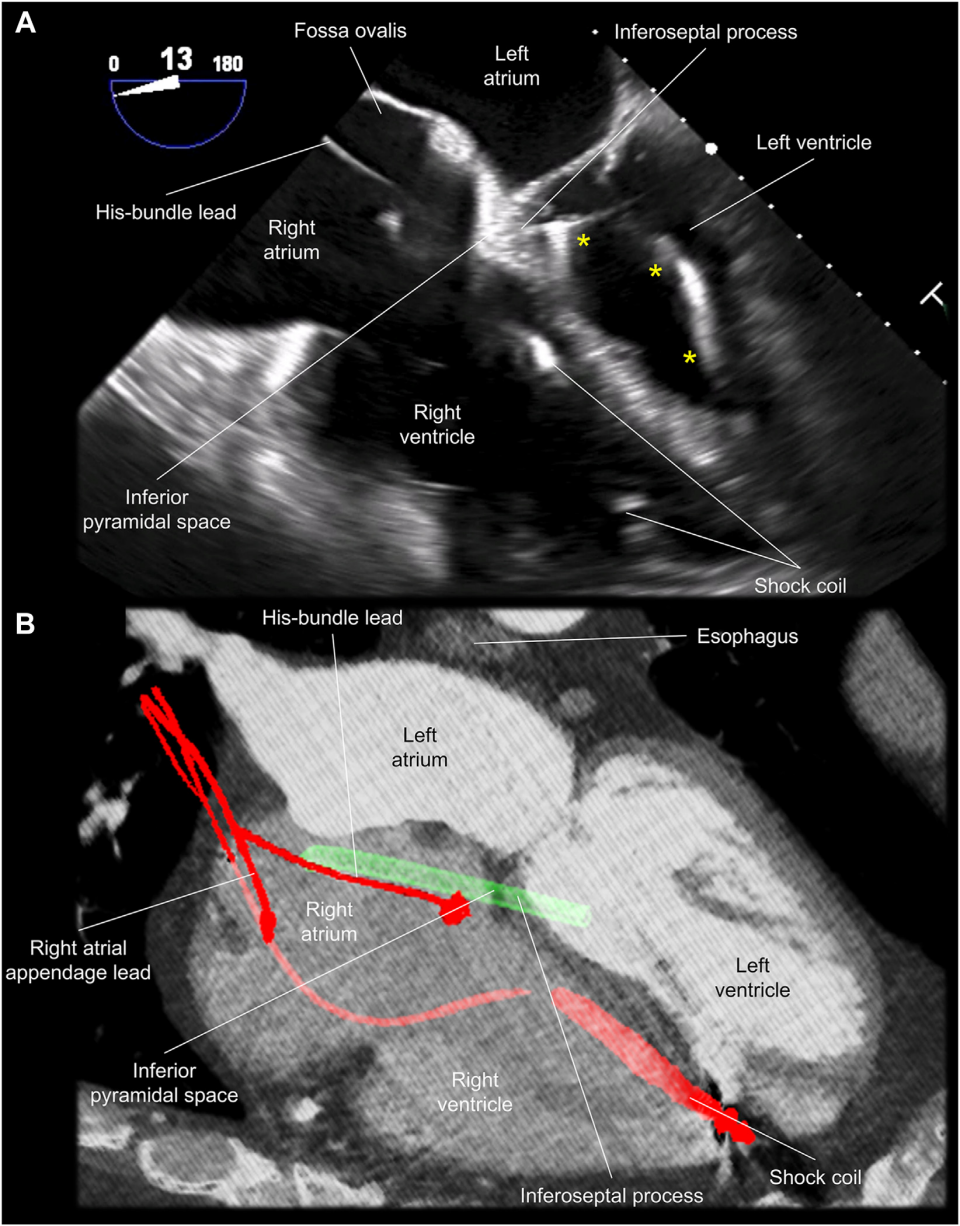
Transesophageal echocardiography. **A:** Transesophageal echocardiography shows the catheter (*asterisks*) placed within the left ventricle after passing through the inferior pyramidal space and inferoseptal process. **B:** replicates the sectional plane of the transesophageal echocardiography **(A)** with a virtual sheath (*green*).

The procedure was guided by the biplane angulation as simulated by pre-procedural CT (Figures 3 and 4) with additional real-time assistance of the electroanatomical mapping system (CARTO3; Biosense Webster Inc., Irvine, CA) and intracardiac echocardiography (SOUNDSTAR Ultrasound Catheter; Biosense Webster Inc.) (Figure 5). The intracardiac echocardiography probe was initially placed in the mid-RA to obtain the home view. Then, with slight anterior flexion and clockwise rotation, the probe is placed near the superior tricuspid vestibule, which was lateral to the His-bundle pacing lead (Figure 5), visualizing the coronary sinus orifice, inferior pyramidal space, inferoseptal process, and basal inferoseptal LV are visualized in a single sector plane (Figure 5). This view was used for the RA-LV access. The procedure was also supported by transesophageal echocardiography with a 4-chamber view at 13° (Figure 6). Through a deflectable sheath (Agilis; Abbott, Abbott Park, IL) and inner catheter (Inquiry Luma-Cath; Abbott), a radiofrequency wire (PowerWire; Baylis medical, Mississauga, ON, Canada) was advanced using radiofrequency energy and delivered into the LV. A microcatheter (GLIDECATH; TERUMO, Tokyo, Japan) was then advanced over the wire, and the wire was exchanged with a stiff wire (Amplatz Super Stiff; Boston Scientific, Marlborough, MA). Then, an 8-mm noncompliant balloon was advanced to the puncture site and inflated, which allowed smooth advancement of the deflectable sheath (Figure 4).

**Figure 7:**
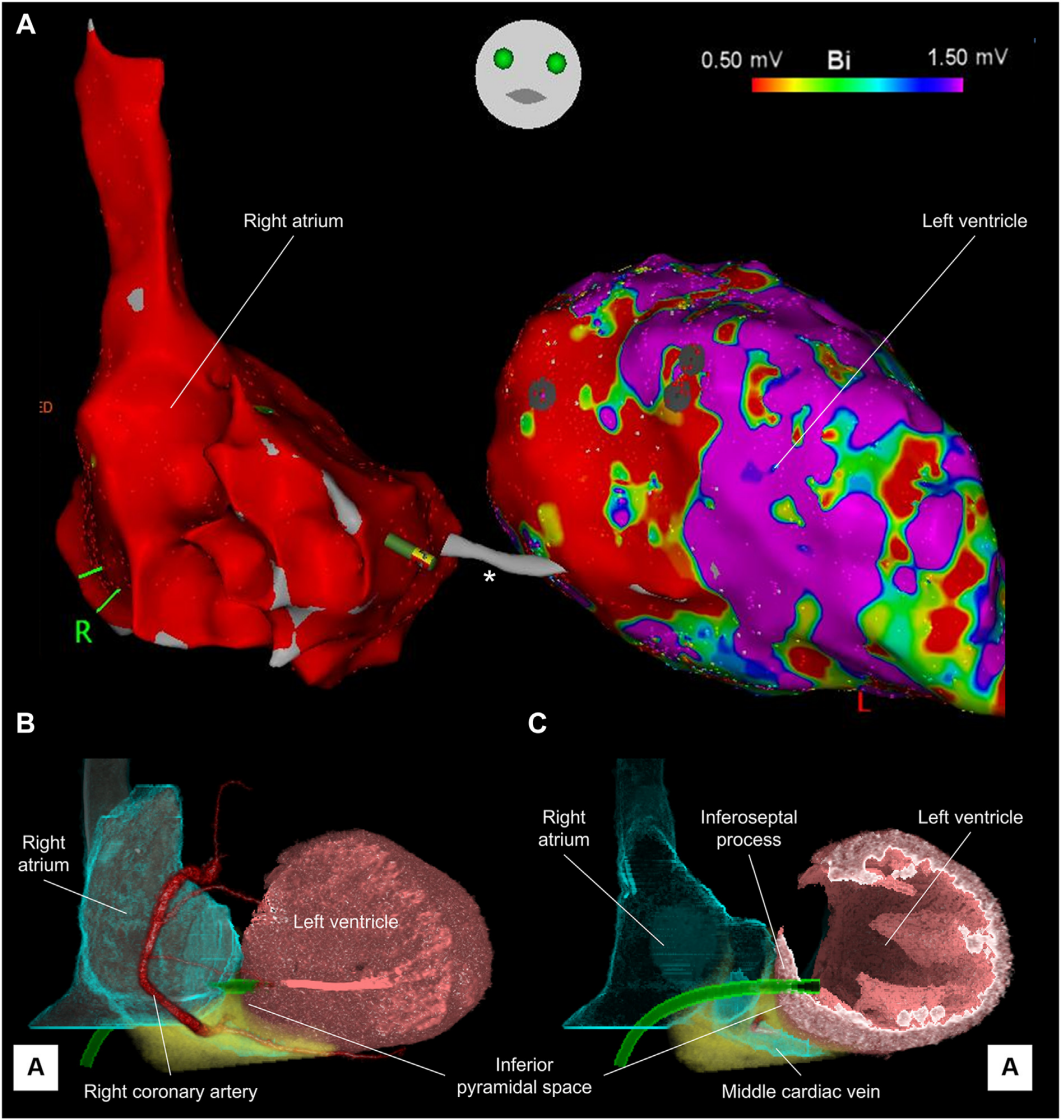
Voltage map of the left ventricle. **A:** After the right atrium to left ventricular puncture, bipolar voltage map of the left ventricle was created, showing dense scar at basal septum. **B and C:** show corresponding volume-rendered images viewed from the frontal direction, reconstructed from the cardiac computed tomography. A virtual sheath is reconstructed in *green*. The special gap between the right atrium and left ventricle in the voltage map (*asterisk*) consists of the right atrial wall, inferior pyramidal space, and inferoseptal process of the left ventricle.

Subsequent catheter ablation was straightforward. Bipolar voltage maps showed dense scars at the basal septal location (Figure 7, Supplemental Movie 2). Pace maps showed that exits of the first and second ventricular tachycardias (Figure 1) were located at the basal septal and anterior portion, respectively. Substrate modification (core-isolation, de-channeling, late-potential ablation) rendered ventricular tachycardia non-inducible. The procedure was terminated without complications. No pericardial effusion and LV to RA fistula were detected after the sheath retrieval.

## Discussion

Patients with aortic and mitral mechanical valves have limited access to the LV.^1^^,^^4^ Regular retroaortic or atrial transeptal approaches should be avoided for such cases. The atrial transseptal approach is technically feasible in a case after transcatheter edge-to-edge mitral repair,^5^ even though available data about potential risk of complications, including a catheter entanglement by the chordae tendineae, dislodge or detachment of clips, and injury of the mitral leaflet, are limited.^6^ In this setting, therefore, potential alternative approaches involve ventricular transseptal access,^7^^,^^8^ transapical access,^9^, ^10^, ^11^ epicardial access,^12^^,^^13^ and the RA-LV puncture via the atrioventricular sandwich.^1^, ^2^, ^3^ At this atrioventricular sandwich, the left ventricular basal inferoseptum, also referred to as the inferoseptal process,^14^ faces to the RA, intervened by the inferior pyramidal space.^15^, ^16^, ^17^, ^18^ The RA-LV puncture through the inferoseptal process has been used in dozens of cases.^1^^,^^2^ Although no fatal complications have been reported, an intramural peri-annular hematoma upon puncture was reported in one patient.^2^

**Figure 8:**
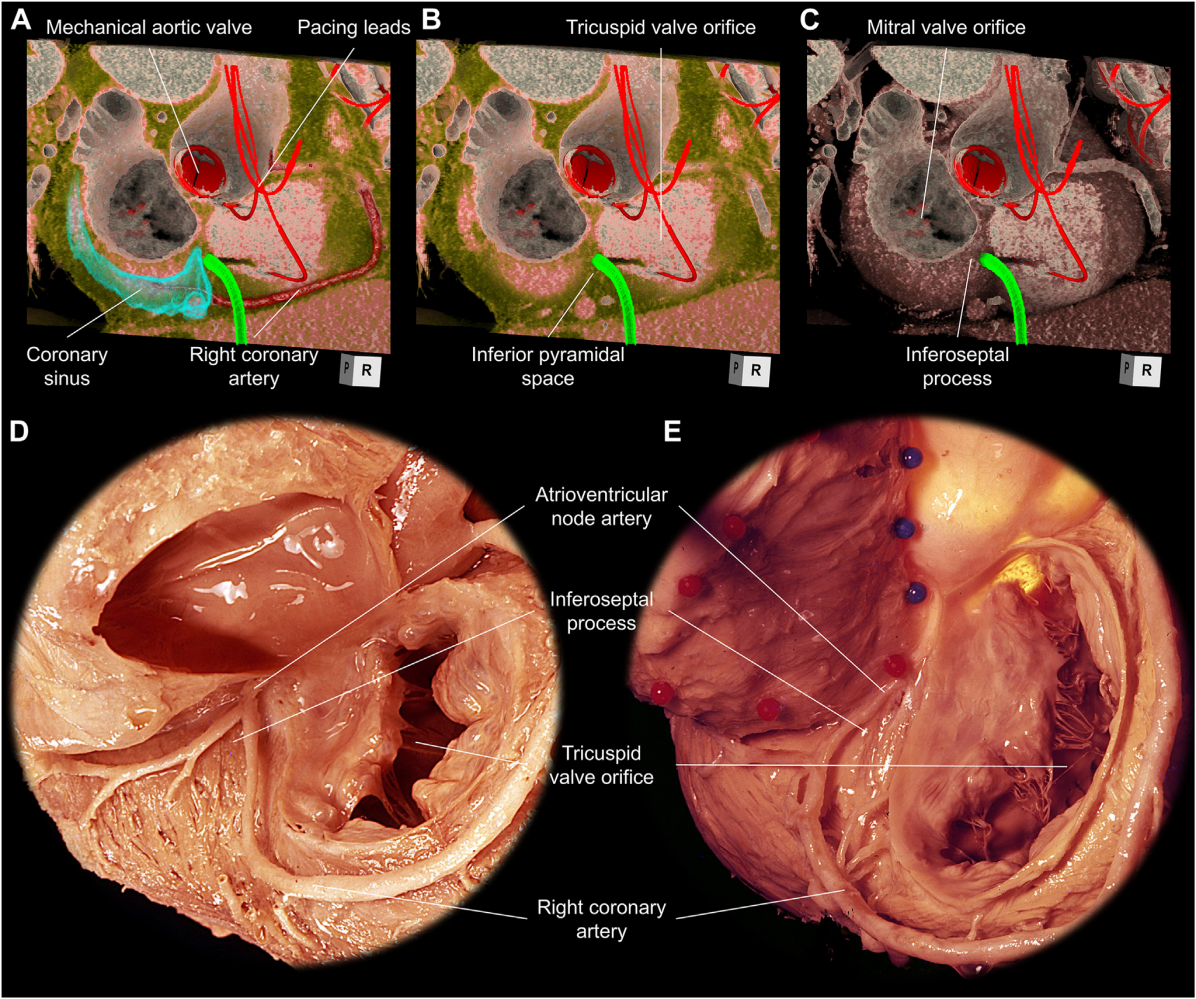
Anatomy of the inferior pyramidal space and inferoseptal process. To puncture from the right atrium to the left ventricle, **A:** the virtual sheath (*green*) needs to be located anterior to the coronary sinus orifice, **B:** pass through the inferior pyramidal space covering the inferoseptal process, and **C:** penetrate the inferoseptal process of the left ventricle. **D:** Reverse U-turn portion of the distal right coronary artery and/or **E:** atrioventricular node artery could be located within this inferior pyramidal space, which should be avoided by pre-procedural evaluation of these structural relationships.

This approach may cause damage to the structures within the inferior pyramidal space.^19^ The inferior pyramidal space is the epicardial fibro-adipose tissue wedging from the inferior crux of the heart toward the central fibrous body, carrying the atrioventricular node on its apex.^15^, ^16^, ^17^, ^18^ It composes the floor of the triangle of Koch and is sandwiched between the RA and LV. In a case with an elongated and eventually tortuous right coronary artery, the distal part of the right coronary artery can show reverse U-turn morphology within the inferior pyramidal space (Figure 8, Supplemental Movie 3).^1^^,^^18^ In addition, the atrioventricular node artery which originates from either the right or left coronary arteries ascends within the inferior pyramidal space.^15^ Thus, any invasive approaches targeting the floor of the triangle of Koch, including the current approach, pose a risk of damaging these structures, which could have fatal consequences. A decision for an alternative approach should be made if the reverse U-turn of the right coronary artery is observed within the inferior pyramidal space. With this regard, pre-procedural evaluation of the inferior pyramidal space and course of the arteries within the space is valid to secure the safety of this procedure.^14^ If cardiac CT is not available, right coronary angiography should be performed and carefully reviewed before the puncture.

The RA-LV puncture is a procedure that can potentially cause epicardial hematoma and/or cardiac tamponade at the time of a sheath removal because the system needs to exit from the RA into the inferior pyramidal space, which is the epicardial region, before re-entering into the LV by penetrating the inferoseptal process. Thus, post-surgical adhesion of the pericardium, which could be expected in potential candidates for this approach, is likely to have some preventive effect for cardiac tamponade. Therefore, we believe that this approach should not be selected for a case without a history of cardiac surgery. Following the RA-LV puncture, a small amount of residual left-to-right shunt has been reported only in one case so far,^2^ which suggests a preventive effect of the multi-layered thick myocardium composing the inferoseptal process for fistula formation, as similar to the ventricular septum puncture.^7^^,^^8^ In addition to pre-procedural planning using cardiac CT, intracardiac echocardiography as well as transesophageal echocardiography (Figures 5 and 6) also played an important and complementary role with their advantage of real-time monitoring and dynamic evaluation of the pertinent structural anatomy, delivery system, and potential complications to secure safety of this procedure.

The location of the heart is known to vary significantly between individuals.^20^^,^^21^ This diversity can directly affect the direction of the RA-LV puncture. Therefore, the biplane angulation needs to be tailored according to the individual patients to keep adequate views to obtain en-face and tangential views of the system. With this regard, pre-procedural simulation with cardiac CT is also of importance.

## Conclusion

Pre-procedural cardiac CT is feasible and useful for the case undergoing the RA-LV puncture. Acquired 3D information can be used not only to evaluate the risk of collateral damage within the inferior pyramidal space, but also to simulate the course of the puncture, and to obtain optimal fluoroscopic angulation to guide the procedure.

## Disclosures

The authors have no conflicts of interest to disclose.
