# Supplementary material for: Pre-procedural simulation of the right atrium to left ventricle puncture through the inferoseptal process using cardiac computed tomography
Source: HeartRhythm Case Rep. 2025 May 23;11(8):767–75. doi: 10.1016/j.hrcr.2025.05.016 (PMC12399163; doi:10.1016/j.hrcr.2025.05.016)
Supplement: Supplementary Data [file mmc4.docx]

**Supplemental movie legends**

**Supplemental Movie 1.** Three-dimensional virtual sheath placed in the left ventricle via the right atrium reconstructed with important landmark structures

**Supplemental Movie 2.** Three-dimensional relationship among the virtual sheath, right atrium, inferior pyramidal space, and left ventricle

**Supplemental Movie 3.** Three-dimensional relationship between the virtual sheath and the inferoseptal process of the left ventricle
